# Trauma‐informed health coaching: A practical guide for COVID‐19 and other disease intervention interviews

**DOI:** 10.1002/puh2.144

**Published:** 2023-12-20

**Authors:** Patricia Mejía, Leona Smith Di Faustino, Alicia DiGiammarino, Thea Sigerman, Sabrina Sanchez, Caitlin Dunn, Jen Tougas, Valerie Kryger, Andrew Maher, Rachel Willard‐Grace

**Affiliations:** ^1^ Department of Family & Community Medicine University of California San Francisco California USA; ^2^ Worker Education & Resource Center, Inc. Los Angeles California USA; ^3^ Teaching and Mentoring Academy, Stanford University School of Medicine Palo Alto California USA; ^4^ Department of Medicine University of California, San Francisco San Francisco California USA; ^5^ Department of Epidemiology and Biostatistics University of California San Francisco California USA; ^6^ Rural Strategic Initiatives Federal Office of Rural Health Policy, Health Resources and Services Administration Rockville Maryland USA; ^7^ Public Health University of California Los Angeles California USA; ^8^ Institute of Global Health Sciences, University of California San Francisco California USA

**Keywords:** disease investigation, health coaching, trauma‐informed care

## Abstract

For people who have experienced psychological trauma, distressing experiences like being notified of exposure to an infectious disease, may trigger a trauma response, a natural, often unconscious, emotional reaction. Disease investigation specialists (DIS), including contact tracers and case investigators, may encounter clients who exhibit trauma responses during outreach calls. In this paper, we describe a novel approach to these calls that combines two evidence‐based approaches: trauma‐informed care and health coaching. These two approaches are put into practice using the HEAR technique, which uses the four steps of Hear, Express Gratitude, Ask, and Respond, to engage with emotionally triggered clients. We provide a series of case vignettes and practical examples of how disease investigation specialists can use the HEAR technique to support clients who may be experiencing trauma. Equipped with this approach, DIS can better engage with the public around existing and emerging infectious diseases, thereby improving both individual health outcomes and public health more broadly.

## INTRODUCTION

As someone living with lupus, Janet has been largely isolated in her studio apartment in a retirement community since the start of the pandemic. She has lost her loved ones to COVID‐19 without being able to be with them in their last days or attend their funerals. Each new surge in cases makes her more despondent about ever getting back to the life she loved and angrier that people around her live as if nothing is happening, not taking precautions to protect themselves and others. When Janet finds out she was exposed to COVID‐19, emotions of anxiety and unresolved grief start to feel overwhelming. And just then, in that moment of intense distress, Janet receives a call from a disease investigation specialist with the local public health department, a stranger to Janet who has lots of questions to ask and advice to give.

As the COVID‐19 pandemic raged, a hidden pandemic of psychological trauma, defined by the American Psychological Association as an emotional response to a distressing event, was spreading across the globe. Many people encountered deeply distressing experiences such as the loss of a loved one, physical violence, or economic instability. Others, especially those isolated from their communities, felt the impact of previous trauma more acutely. Historically marginalized groups, including older adults, people with chronic health conditions, those with low socioeconomic status, the underinsured, and those from diverse racial and ethnic backgrounds, have borne a higher burden of COVID‐19 infections and deaths [1, 2, 3, 4, 5] and are likely, therefore, to also bear a greater psychological burden.

Distressing experiences can trigger a trauma response, which is a natural and often unconscious emotional reaction. When the body perceives a threat, it reflexively sends chemicals into the bloodstream that activate the sympathetic nervous system, which controls the fight, flight, or freeze responses. If trauma responses are repeatedly activated, the body enters a sustained survival mode with increased reactivity to even seemingly small triggers. A person exhibiting a trauma response is less likely to be able to engage in collaborative conversations with health outreach workers, including disease investigation specialists (DIS), who work to interrupt the transmission of infectious diseases, including sexually transmitted diseases, tuberculosis, and COVID‐19 [6].

DIS interrupt the spread of transmissible diseases by (1) identifying people who may have been exposed to the virus and (2) helping those who test positive to receive treatment quickly. Due to the nature of this work, DIS, including case investigators (CI) and contact tracers, often speak with people during periods of increased vulnerability. The stress of testing positive, being notified of an exposure, or navigating illness, quarantine, and isolation, can trigger a trauma response, especially in those already navigating psychosocial stressors. Prolonged exposure to people exhibiting trauma responses can, in turn, lead to vicarious trauma and burnout for DIS [7].

The California Virtual Training Academy (VTA+), a collaboration between University of California San Francisco, University of California Los Angeles, and the California Department of Public Health, developed a multidisciplinary training that uniquely combines the principles of two evidence‐based approaches, Trauma‐informed Care and Health Coaching, to support pandemic response workers and other DIS across the State of California. This model has the potential to be used widely in the context of public health disease investigation and intervention. In this paper, we use a series of COVID‐19 case investigation vignettes to provide practical examples for application of the model in DIS work.

## TRAUMA‐INFORMED CARE

*As a CI focused on high‐risk congregate settings, you call Janet to tell her that she has tested positive for COVID‐19. Janet is irate when she hears the news. “How dare you start lecturing me about isolation and testing! You people just stopped even trying! Nobody's masking. Nobody's doing anything. The health department is just acting like nothing's happening! Of course this is going to blow up!” When you try to calm her down, she snaps, “Do you have ANY idea what it's like to sit in your house for two years and stare at the wall, and then get COVID because the health department can't even be bothered to do their job?! Of course you don't!”*

In this example, Janet is clearly upset, and the CIs may take the anger personally. In fact, Janet may be exhibiting a trauma response. She is not necessarily combative due to anything the DIS said, but rather hyper‐reactive due to the uncertainty and stress of the pandemic.

If the CI is trained to examine Janet's reaction through a trauma‐informed lens, they will recognize that Janet's positive test result triggered a fight response in which she yelled angrily at the DIS. If Janet had sought a quick exit from the uncomfortable conversation by rushing to end the call, she would have been demonstrating a flight response. Simply falling quiet would have been an example of a freeze response.

Trauma‐informed care integrates best practices to shape the way in which care providers and organizations prepare for and respond to trauma responses in their clients so as to actively avoid inflicting any additional harm. The tenets of trauma‐informed care are safety, trustworthiness and transparency, peer support, collaboration, empowerment, and humility and responsiveness [8] (see Table 1).

**TABLE 1:** Tenets of trauma‐informed care with practical examples for disease intervention interviews.

Principle	Definition	Example
Safety	Promoting feelings of physical and psychological safety	Acknowledging Janet's remarkable efforts to keep herself physically safe from infection, as well as her struggle to feel safe psychologically
Trustworthiness and transparency	Conducting interactions with the goal of being forthright and truthful as a basis for establishing trust with clients	Being forthright about what the DIS can or cannot offer
Peer support	Connecting clients with other survivors of distressing experiences, who have lived experiences to share	Connecting Janet to other people who have been similarly isolated by the pandemic
Collaboration	Leveling of power and decision‐making	Asking about Janet's goals and how the DIS can best support her
Empowerment	Building on the strengths of clients, supporting them in their decisions and authority in their lives	Recognizing Janet's resourcefulness and strength through a very difficult period
Cultural humility and awareness of cultural, historical, and systemic oppression	Being aware of historical harms and of one's own limitations in understanding the suffering of others	Acknowledging that the DIS does NOT know what Janet has suffered

## HEALTH COACHING

Health Coaching is a set of evidence‐based skills designed to help people build knowledge, skills, and confidence to make positive changes toward improving their health [8] (see Table 2). These skills are rooted in similar principles to those outlined in Table 1, including that engaging with clients, who are experts in their own lives, requires a client‐centered, strength‐based, collaborative approach. The CI can be client‐centered by putting their own agenda for the call on hold and seeking to better understand Janet's experience. They can be strength‐based by recognizing Janet's expertise, her community‐based resources, and her incredible motivation to protect her health as demonstrated by having stayed home for two full years. They can be collaborative by seeking to identify Janet's goals, share experiences, and offer information or resources to support Janet through isolation.

**TABLE 2:** Health coaching skills.

Skill	Definition	Example
Set the Agenda	Identify what both the DIS and the contact would like to cover in the conversation and agree on how best to use the time during the call	“What questions or concerns would you like to be sure we talk about in our call today?”
Ask Tell Ask	Assess and build on what the client already knows and is motivated to do	“You have made remarkable efforts to stay safe during the pandemic. What do you know about how to prevent the spread of the virus?”
Action Plan	Work with the client to create specific, short‐term plans to take them one‐step closer to meeting a larger goal	“It sounds like you are interested in talking to other people who have had similar experiences. Would it be helpful to work together on a plan for making that happen?”

## APPLICATION OF THE TRAUMA‐INFORMED HEALTH COACHING MODEL

DIS frequently have defined tasks that they need to accomplish during the course of an interview (e.g., elicit contacts, schedule a vaccination appointment) or information that they need to impart (e.g., instructions for testing, isolation, and quarantine). However, when a client exhibits a trauma response, it is unlikely that they will effectively take in or share information unless the DIS prioritizes connecting with them.

In the Trauma‐Informed Health Coaching approach, DIS learn how to put the principles of trauma‐informed care and health coaching skills into practice using the HEAR (hear, express appreciation/gratitude, ask, and respond) technique. The HEAR technique provides an easy‐to‐remember method to validate a client's experiences, recognize their strengths, and offer support in a way that is collaborative and focused on the needs of the individual [9].

## THE HEAR TECHNIQUE STANDS FOR HEAR, EXPRESS APPRECIATION, ASK, AND RESPOND

**HEAR**: Use open‐ended questions and reflective statements to better understand the client's concerns. Open‐ended questions invite further conversation, a marked contrast to many people's natural desire to curtail an uncomfortable conversation. A reflective statement seeks to rephrase and acknowledge what the client is saying or how they are feeling:

*When Janet expresses anger [the fight response], the DIS acknowledges those feelings and invites additional information*.

DIS*: This is really upsetting. Can you tell me more?*

*Janet: You have NO idea what it's been like. I've barely seen the street outside my house for two years. My neighbors have even stopped coming by because they know I won't invite them in. And now you're telling me that after all that, all that I've been through, I got this damned virus anyway?!?*

DIS*: You're absolutely right that I can't know what your experience has been like. You worked really hard to stay safe. This doesn't seem fair*.

*Janet: Well, what really makes me angry is how everyone's pretending the pandemic is over. No one is even masking in the hallways anymore. People are still dying! My best friend in Chicago just died from COVID. He protected himself for two years, but he got it anyway. It's like even the health departments have just stopped trying, and nobody cares anymore*.

DIS*: It feels like the world is going back to business as usual, but for you, this is serious*.

Janet*: Yeah, I've got lupus, so it's life or death for me. It's like it doesn't matter to the rest of the world what happens to me*.

Notice that as long as Janet continues to feel heightened emotions, the DIS remains in the “HEAR” phase to acknowledge Janet's feelings and understand the source of her frustration more deeply.

Linking back to the principles of trauma‐informed care (Table 1), the DIS creates psychological safety by actively seeking to understand Janet's feelings and concerns. The DIS also demonstrates trustworthiness and cultural humility by acknowledging their own limited capacity to understand Janet's lived experience and providing space for her to share more.

**Express appreciation/gratitude**: Thank the client for the efforts they are making. In the context of the pandemic, many people have gone to great lengths to protect themselves and others around them, stay informed, and follow shifting public health guidance. They rarely receive thanks for their efforts. DIS can offer much needed gratitude and validation for the efforts they have made. For example,

DIS*: You've worked so hard to keep safe …. And to keep others safe, too. Thank you for that*.

Within the principles of trauma‐informed care (Table 1), the DIS’ expression of gratitude is *empowerment* in that it explicitly identifies Janet's strengths and the efforts she has made.

**Ask**: Having conveyed respect and validation in the first two steps of the HEAR Technique, ask what would be most helpful to the client and ask for permission to provide information or support:

DIS: *What can I do to support you right now?*

*Janet: Well, I'm pretty much set for isolation, since that's basically what I've been doing for the last two years of this damn pandemic. You know what would really help? A punching bag to let out my anger. Honestly, it would be great to have someone that I could just talk to or yell at without worrying about their feelings*.

DIS: *It sounds like you've been through a lot over the last few years. It would be great for you to have an outlet*.

Janet: *I guess I've been venting to you on this call. Thanks for listening*.

DIS: You *are welcome, Janet. Would it be okay to share some resources or perhaps a peer support group that you can reach out to if you need more help?*

Janet*: I didn't realize there were groups like that. Yes, I'd love to take down the number or website*.

In “Ask” the DIS demonstrates the trauma‐informed care principles of the following:

- *Collaboration* by drawing out Janet's own goals in order to tailor the resources provided.
- *Empowerment* by asking permission to share resources, thereby allowing Janet to remain in control of the conversation.
- *Peer Support* by connecting Janet with others who have a similar lived experience.

**Respond**: Offer new information or resources, tailored to the needs of the client. “Respond” frequently includes the tasks that DIS are responsible for, such as eliciting contacts or going over isolation guidelines.

DIS: *There are a few options for places to reach out to that I can share with you before the end of this call. For example, along with the peer support groups there is a state hotline. I'd also like to make sure you know about what symptoms might mean you need to seek help. I'd like to make sure you know how to get tested and to find out who you've been in contact with. Would that be okay?*

By using the HEAR Technique, the DIS combines trauma‐informed care principles with health coaching skills to slow down the conversation (phases H, E, & A). They understand, address, and acknowledge Janet's needs before moving to their own agenda in the “Respond” phase.

## IMPLICATIONS FOR POLICY AND PRACTICE

After getting off the call with Janet, you are feeling shaky. Janet's story reminds you of your uncle, and how hard he tried to protect himself and your aunt before he ultimately caught COVID‐19 and died in the ICU just a month before the vaccines came out. You reach out to your supervisor by email to say that you had a hard call and to ask if you can take off the last few hours of your shift today.

Providing Trauma‐Informed Health Coaching can be deeply satisfying in as much as it can allow DIS to more effectively connect with and offer support to community members. When clients are in a position to share their experience, they are more likely to be able to take in new information and follow guidelines. At the same time, encountering the trauma responses of clients can cause distress for the DIS seeking to support them. Sustainable implementation of the proposed model requires not only changes to a DIS’ individual approach, but also structural changes to the system (Table 4).

## CONCLUSION

Based on our experience training over 12,000 DIS in California during the COVID‐19 pandemic, we distilled two evidence‐based approaches, trauma‐informed care and health coaching into a scalable model for public health responders to more effectively and empathetically engage with clients. The HEAR Technique, as outlined in Table 3 , provides steps that equip DIS to engage with the public around existing and emerging infectious diseases, thereby improving both individual health outcomes and public health more broadly.

**TABLE 3:** The HEAR technique.

Step	Definition	Example
Hear	Ask open‐ended questions to invite the person to share their thoughts and feelings. Listen carefully and reflect back the content or feeling of what they share	“This is really upsetting. Can you tell me more?”
Express gratitude	Thank the person for sharing their thoughts or concerns. When someone shares their concerns or their skepticism with us, we should consider it a gift	“You've worked so hard to keep safe …. And to keep others safe, too. Thank you for that”
Ask	Ask what would be most helpful to the client and ask for permission to provide information or support	“What can I do to support you right now?” “Would it be okay to share some resources or perhaps a peer group that you can reach out to if you need more help?”
Respond	Summarize what you've heard. Ask permission to share key information or discuss next steps	“Let me see if I'm hearing this correctly .…” “There are a few options for places to reach out to that I can share with you before the end of this call. I'd also like to make sure you know about what symptoms might mean you need to seek help. I'd like to make sure you know how to get tested and to find out who you've been in contact with. Would that be okay?”

**TABLE 4:** Sustainable trauma‐informed approach: levels of change and action steps.

Level of change	Action steps
Individual	Increase one's own knowledge about and skills in Trauma‐Informed Health Coaching and the HEAR technique Advocate for use of the Trauma‐Informed Health Coaching within the organization Apply trauma‐informed care and health coaching techniques in calls/community outreach
Organizational	Assess current practices and policies, awareness of, and existing capabilities for trauma‐informed care, health coaching, and the HEAR technique Provide training across the organization to increase capacity to respond to clients exhibiting a trauma response Build supervisor understanding of trauma‐informed care, HC, and vicarious trauma so they can mentor and support frontline staff Validate and address vicarious trauma experienced by DIS Adjust evaluation metrics so that DIS are not penalized for the lower number of clients reached when using the Trauma‐Informed Health Coaching model
Community	Develop relationships with community partners in order to Train DIS in Trauma‐Informed Health Coaching Create a network of support for people experiencing psychological trauma Create opportunities for knowledge sharing and evaluation of Trauma‐Informed Health Coaching in the community

## AUTHOR CONTRIBUTIONS

*Conceptualization; writing—original draft; writing—review and editing*: Patricia Mejia. *Conceptualization; writing—original draft; writing—review and editing*: Leona Smith Di Faustino. *Conceptualization; writing—original draft; writing—review and editing*: Alicia DiGiammarino. *Conceptualization; writing—review and editing*: Thea Sigerman. *Conceptualization; writing—review and editing*: Sabrina Sanchez. *Conceptualization; writing—review and editing*: Caitlin Dunn. *Conceptualization; writing—review and editing*: Jen Tougas. *Conceptualization; writing—review and editing*: Valerie Kryger. *Conceptualization; writing—review and editing*: Andrew Maher. *Conceptualization; writing—original draft; writing—review and editing*: Rachel Willard‐Grace.

## CONFLICT OF INTEREST STATEMENT

The authors have no conflicts of interest to declare.

## FUNDING INFORMATION

This work was supported in part by the California Department of Public Health, Agreement Number 19‐11102

## Data Availability

Data sharing not applicable—no new data generated.

## References

[1] Fouad MN , Ruffin J , Vickers SM . COVID‐19 is disproportionately high in African Americans: this will come as no surprise …. Am J Med. 2020;133(10):e544‐e545. 32442510 10.1016/j.amjmed.2020.04.008 PMC7237375

[2] Hooper MW , Nápoles AM , Pérez‐Stable EJ . COVID‐19 and racial/ethnic disparities. JAMA. 2020;323(24):2466‐2467. 32391864 10.1001/jama.2020.8598 PMC9310097

[3] Harris OO , Leblanc N , McGee K , Randolph S , Wharton MJ , Relf M . Alarm at the gate‐health and social inequalities are co‐morbid conditions of HIV and COVID‐19. The Journal of the Association of Nurses in AIDS Care: JANAC. 2020;31(4):367. 32568762 10.1097/JNC.0000000000000190 PMC8272948

[4] Yancy CW . COVID‐19 and African Americans. JAMA. 2020;323(19):1891‐1892. 32293639 10.1001/jama.2020.6548

[5] Mackey K , Ayers CK , Kondo KK , et al. Racial and ethnic disparities in COVID‐19‐related infections, hospitalizations, and deaths: a systematic review. Ann Intern Med. 2021;174(3):362‐373. 33253040 10.7326/M20-6306 PMC7772883

[6] Centers for Disease Control and Prevention . Disease Intervention . 2021. Division of STD Prevention, National Center for HIV, Viral Hepatitis, STD, and TB Prevention, Centers for Disease Control and Prevention. https://www.cdc.gov/std/projects/disease‐intervention/default.html#print

[7] Substance Abuse and Mental Health Services Administration . SAMHSA's Concept of Trauma and Guidance for a Trauma‐Informed Approach . *HHS Publication No. (SMA) 14‐4884. Rockville, MD: Substance Abuse and Mental Health Services Administration*. 2014.

[8] Ghorob A . Health coaching: teaching patients how to fish. Fam Pract Manag. 2013;20(3):40‐42. 23939739

[9] DiGiammarino A . Try this new technique when talking to vaccine skeptics. *KevinMD*. 2021. Accessed October 15, 2021. https://www.kevinmd.com/2021/08/try‐this‐new‐technique‐when‐talking‐to‐vaccine‐skeptics.html

